# Siltation and tidal effects: a comprehensive, clinical, and didactic approach to reasoning about vertebrobasilar hypoflow conditions

**DOI:** 10.1016/j.bjorl.2026.101822

**Published:** 2026-04-29

**Authors:** Arlindo Cardoso Lima Neto, Raquel Mezzalira, Roseli Saraiva Moreira Bittar

**Affiliations:** a Universidade de São Paulo, Department of Otorhinolaryngology, São Paulo, SP, Brazil; b Universidade de Campinas, Faculdade de Ciências Médicas, Discipline of Otorhinolaryngology, Head and Neck, Campinas, SP, Brazil

**Keywords:** Vertebrobasilar insufficiency, Cerebrovascular circulation, Vertigo dizziness, Hemodynamics

## Abstract

**Objectives:**

To propose a clinically applicable model for understanding vertebrobasilar hypoperfusion by categorizing its causes into two mechanisms: the siltation effect, related to local vascular obstruction, and the tidal effect, related to systemic reduction in posterior circulation blood flow.

**Methods:**

A non-systematic narrative review was conducted using selected literature in the fields of neurotology, vascular neurology, and cardiovascular medicine. The analogy of coastal rivers affected by sedimentation and tides was used to explain the proposed classification. Clinical examples were drawn from studies on vertebral artery narrowing, subclavian steal syndrome, dolichoectasia, small vessel pathology, orthostatic hypotension, cardiac disorders, anemia, and abnormal blood pressure regulation.

**Results:**

The siltation effect includes conditions that block or reduce blood flow within the vertebrobasilar system, such as atherosclerosis, vessel compression, and dissection. The tidal effect includes disorders that globally impair perfusion, such as heart failure, low blood pressure, reduced oxygen delivery, and circadian blood pressure extremes. Combined situations, such as arterial hypotension, vessel stenosis, sleep disorders and migraine are common and may increase the risk of transient ischemic events or stroke.

**Conclusion:**

The distinction between siltation and tidal effects provides a useful clinical reasoning model for diagnosing and managing patients with symptoms suggestive of vertebrobasilar hypoflow, including vertigo, dizziness, and transient brainstem symptoms. Recognizing the frequent coexistence of these mechanisms reinforces the need for a comprehensive, multidisciplinary approach to ensure accurate diagnosis and guide appropriate therapeutic decision-making.

**Level of evidence:**

4.

## Highlights

• Classification based on the analogy of sedimentation and tides.
• Siltation effect refers to conditions that block blood in the vertebrobasilar system.
• Tidal effect includes disorders that globally impair perfusion.
• Recognizing these mechanisms guide therapeutic decision-making.

## Introduction

Vertigo, dizziness, transient visual loss, diplopia, syncope, unexplained falls, dysphagia, and dysarthria are some common symptoms of Vertebrobasilar (VB) hypoflow, which can result from a variety of underlying conditions.^1^^,^^2^ The Bárány Society recently published the Vascular Vertigo and Dizziness (VVD) diagnostic criteria, encompassing strokes, Transient Ischemic Attacks (TIAs), isolated labyrinthine ischemia or hemorrhage, and Vertebral Artery Compressive Syndrome (VACS).^3^ Although this document was crucial in defining these concepts, the etiologies listed in the differential diagnoses were not emphasized.

Other conditions that can mimic VVD symptoms include subclavian steal syndrome (SSS), early stages of small vessel disease (SVD), severe anemia, arrhythmia, heart failure, orthostatic hypotension, and other dysautonomia syndromes.^4^, ^5^, ^6^ Extreme dipping (i.e. a marked nocturnal drop in blood pressure), commonly seen in the context of systemic hypertension, may also contribute to VB hypoflow.^7^

**Fig. 1:**
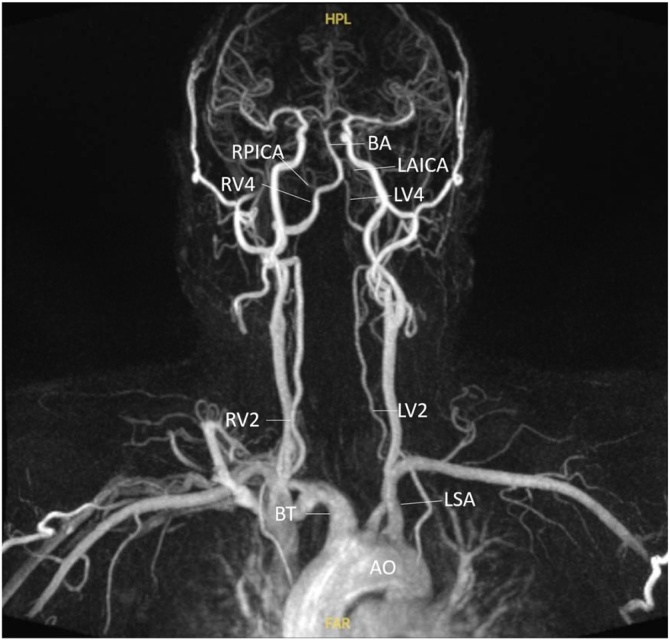
MR angiography of a patient with hypoplasia of the left vertebral artery. AO, Aorta; BT, Brachiocephalic Trunk; LSA, Left Subclavian Artery; RV2, Second segment of the Right Vertebral artery; LV2, Second segment of the Left Vertebral artery; RV4, Fourth segment of Right Vertebral artery; LV4, Fourth segment of Left Vertebral artery; RPICA, Right Posterior Inferior Cerebellar Artery; LAICA, Left Anterior Inferior Cerebellar Artery; BA, Basilar Artery. Note de asymmetry between RV2 (3.8 mm) and LV2 (1.8 mm) [1:2.1], as well as between RV4 (3.6 mm) and LV4 (1.8 mm) [1:2] due to the hypoplasia of the left vertebral artery.

Analogous to large rivers near coastal areas, the anatomical proximity between the Vertebral Arteries (VA) and the aorta increases the susceptibility of the VB circulation to systemic hemodynamic influences (Fig. 1). Moreover, several studies have explored how VB flow is modulated by vascular geometry, suggesting that certain anatomical configurations may predispose to or exacerbate pathological flow states.^8^, ^9^, ^10^, ^11^, ^12^

In this non-systematic review, we gathered some of the most comprehensive and cited publications about the conditions that cause VB hypoflow. The goal is to train clinicians to reason pathophysiologically and conduct more comprehensive etiological investigations. The conceptual framework is based on an analogy with tide-affected rivers, which are prone to siltation and oscillatory flow dynamics.

## The siltation effect

The vast majority of rivers experience siltation, but only those influenced by the seas have tidal cycles. Examples of such phenomena include the Amazon River, the Qiantang River, the Trent, the River Thames, the Batang, and the Seine.^13^ Just as silting in rivers makes them more shallow and decreases their flow, in this analogy, we gather diseases linked to blood block as “siltation conditions” in VB territory (Table 1).

**Table 1:** Conditions linked to the siltation effect, the tidal effect, or both.

Local Conditions “Siltation Effect”	Systemic Conditions “Tidal Effect”	Overlapping
VB Ischemic Stroke	Heart failure	A mix of two or more
VB TIA	Acute myocardial infarction	Obstructive sleep apnea
Labyrinthine ischemia	Arrhythmia	
Vertebral artery compressive syndrome	Other cardiac blood-pumping failure conditions	
Subclavian steal syndrome	Orthostatic hypotension	
Vertebrobasilar stenosis	Drug-related hypotension	
Vertebral artery hypoplasia	Small vessel white matter disease	
Vertebrobasilar dissection	Other dysautonomic syndromes	
Vertebrobasilar dolichoectasia	Lung diseases	
Other thromboembolic diseases	Anemias	
Migraine		
Reversible cerebral vasoconstriction syndrome		
Other vasoespastic diseases		

VB, vertebrobasilar; TIA, transiente ischemic attack.

### Cerebral and labyrinthine ischemic events

The ischemic stroke certainly epitomizes this group, particularly when it results from an obstruction in a large vessel.^3^ Small vessels are more commonly involved in lacunar strokes and TIAs.^14^ When a blockage occurs in the internal auditory artery or any of its branches, the patient may exhibit symptoms of labyrinthine ischemia ‒ either auditory or vertibular ‒ depending on which branches are affected. The inner ear ischemia is a presumptive diagnosis, as it is practically impossible to confirm without a pathological study.^3^^,^^15^

The current concept of VACS encompasses the previous bow hunter’s syndrome, rotational VA syndrome, rotational VA compression syndrome, and rotational VA occlusion syndrome.^3^^,^^16^ The diagnosis is confirmed in cases of vertigo, with or without tinnitus, triggered by a sustained eccentric neck position. It must be supported by the presence of nystagmus and documentation of VA compression using dynamic angiography, or demonstration of decreased blood flow using transcranial Doppler ultrasound.^3^

### Subclavian Steal Syndrome (SSS)

Another example of the “VB siltation effect” is SSS. This rare yet well-known phenomenon occurs when an occlusive lesion of the proximal subclavian artery causes the VA to reverse its flow.^4^^,^^17^ The most common cause is atherosclerosis, which gives rise to stenosis and occlusion in more then 90% of the cases. Because the left subclavian artery has a more prominent angle at its origin, turbulent flow will accelerate atherosclerosis and account for more than 80% of the cases.^17^^,^^18^ In addition to VB hypoflow symptoms, some patients report intermittent claudication, paresthesia, or weakness in the upper extremities, predominantly in the left arm.^4^ However, only 5.3% of patients with sonographic evidence of SSS experience symptoms.^17^^,^^18^ The most important predicting factor for symptomatic presentation of SSS seems to be the magnitude of arterial blood pressure difference between upper extremities.^18^ Another controversy is the finding of the reversal phenomenon in patients without subclavian artery stenosis but with either a hypoplastic VA or significant proximal stenosis of the vessel. A hypoplastic VA is assumed to cause bidirectional flow due to increased flow resistance within the vessel. In contrast, proximal VA stenosis would result in episodes of reverse flow due to collaterals, such as the spinal arteries.^19^

### Vertebral artery hypoplasia

VA obstructions of more than 50% are associated with an increased risk of stroke.^3^ But the role of VA hypoplasia has been clarified recently. In a meta-analysis including a total of 3,875 acute ischemic stroke patients, a significantly higher probability of the presence of VA hypoplasia was found in VB stroke patients compared to patients with anterior circulation strokes (risk ratio¼ 2.12, 95% CI: 1.60–2.82, p < 0.001).^9^ The majority of the enrolled studies considered a vessel diameter of 2.0 mm or less or a ratio of 1:1.7 to the contralateral side to be hypoplastic^9^ (Fig. 1).

### Arterial dissection

Another significant vascular disease of the VB system is dissection, which is a considerable cause of ischemic stroke in young and middle-aged patients, accounting for 10% to 25% of cases.^20^ It mostly affects the VA, resulting in blockage, aneurysm formation, and subsequent thromboembolic effects.^21^

### Geometric aspects

The configuration of the VB system, in which two arteries merge into a single vessel, is unique in the human anatomy.^10^ When VAs of similar magnitude join, blood flowing into the basilar artery travels nearly parallel to the vessel wall without mixing. In contrast, if the VAs have significantly different diameters, the flow of the basilar artery is disturbed by the formation of swirling and secondary flows.^11^ There are still few prospective clinical studies supporting the risks associated with the VB system's different geometric configurations. Although prospective clinical studies on VB geometry are limited, Qu et al. followed 420 patients with VB stroke for one year and found that certain configurations ‒ such as bending and VA hypoplasia ‒ were significantly associated with large-artery atherosclerosis, recurrent stroke or TIA, and mortality. The authors emphasized the importance of considering vascular geometry when assessing stroke risk in the VB territory.^8^

### VB Dolichoectasia

However, stretching and excessive dilation of the arteries can also create disturbances in the flow of blood through the basilar and vertebral arteries.^22^, ^23^, ^24^

Smoker et al. established diagnostic standards for the basilar artery using cranial CT.^25^ Subsequently, Ubogu et al. expanded the analysis to utilize MRI and included the vertebral arteries.^24^^,^^25^ VB ectasia is defined as a vessel diameter ≥4.5 mm at any point along its course.The MRA standards raised by Ubogu define extension by MRA as the length of basilar artery greater than 29.5 mm or the vertical distance from the connection of the basilar artery starting point and a bifurcation point greater than 10 mm. For vertebral arteries, if the length is greater than 23.5 mm, or at any point the vertical distance from the connection of the skull entry point and the basilar artery starting point is greater than 10 mm, it is considered extension.^23^^,^^24^ However, the variations between genders and populations are not yet fully established.^23^

The etiology of dolichoectasia has been associated with many diseases such as systemic arterial hypertension, atherosclerosis, collagen-related diseases, polycystic kidney disease, and sickle cell anemia.^23^^,^^26^^,^^27^ One possible mechanism behind this disease is an imbalance in the activity of metalloproteinases and proteinases in the connective tissue of arterial walls. This imbalance leads to abnormal vascular remodeling and defective connective tissue formation in arterial walls.^27^

Clinical manifestations of VB dolichoectasia vary widely, from asymptomatic status to exertional headache, or even to fatal ischemic stroke or hemorrhage.^22^ Considering non-life-threatening symptoms, nearly 40% of patients can develop disorders related to the VIII cranial nerve. These symptoms are predominantly related to two mechanisms: thrombotic-ischemic vascular mechanisms, in which the swirling flow pattern promotes thrombosis and microemboli, and compressive mechanisms, in which the elongated and tortuous arteries directly displace surrounding tissues, including cranial nerves and the brainstem. This nerve compression can mimic tumors of the cerebellopontine angle, presenting with retrocochlear findings on audiometry and auditory brainstem potentials.^28^

### Migraine

Emerging evidence supports a link between migraine and stroke, especially in young women.^29^^,^^30^ While migraine can directly cause stroke in the form of migrainous infarction, it may also act as an independent risk factor.^29^^,^^31^ Although the precise mechanism remains unclear, inflammatory and endothelial alterations ‒ as well as associations with patent foramen ovale, oral contraceptive use, and smoking ‒ are thought to play roles. Vasoconstrictors such as triptans and ergot derivatives, commonly used in treatment, may also contribute.^29^

An important differential diagnosis is Reversible Cerebral Vasoconstriction Syndrome (RCVS), characterized by thunderclap headaches ‒ often accompanied by other neurologic symptoms such as vertigo ‒ and multifocal cerebral artery narrowing that typically resolves within three months.^32^^,^^33^

## The tidal effect

In this metaphorical and didactic approach, "the tidal effect" refers to conditions in which blood availability decreases in the VB system. In such cases, the underlying cause of flow deprivation is diseases external to the VB system that impair adequate blood supply.

### Cardiac conditions

Dizziness and vertigo are common symptoms in patients with heart failure, acute myocardial infarction, or arrhythmias.^34^^,^^35^ Heart disease may be a differential diagnosis for VVD, or it may be an associated factor. Therefore, a detailed cardiac workup is required.^3^^,^^14^ Cardiac pump failure syndromes are prototypical examples of the “low tidal effect”. Among these, sporadic cardiac arrhythmias pose the greatest diagnostic challenge. The Holter monitor is the gold standard for diagnosing arrhythmias, but it is nondiagnostic in more than 90% of syncope-related cases.^36^ Loop recorders can be used to extend the monitoring period and increase the probability of obtaining a symptom-rhythm correlation.^37^ However, within this conceptual framework, arrhythmias that cause symptoms of VB hypoflow due to thromboembolism, such as atrial fibrillation, must be considered “siltation”, not “tidal effect”.^38^

### Hypotensive conditions

Systemic arterial hypotension is defined as a Blood Pressure (BP) below 90/60 mmHg.^39^^,^^40^ Nontraumatic, symptomatic episodes are more prevalent among older adults. In contrast, resting BP that are typically not clinically concerning are often found in physically active and otherwise healthy individuals.^39^ A significant number of hospital admissions involving hypotension are drug-related. Several commonly used medications may lower BP, including antihypertensives (angiotensin-converting enzyme inhibitors, beta-blockers, and diuretics), neuroleptics, alpha-1 blockers for benign prostatic hyperplasia, benzodiazepines, opioids, and antidepressants.^40^ These agents should be carefully managed in the presence of VB hypoflow symptoms.

Orthostatic hypotension is defined by a reduction in systolic (>20 mmHg) and/or diastolic (>10 mmHg) BP within 3-minutes upon standing from sitting or during head-up tilt test.^41^ The pathophysiology behind this phenomenon is dysautonomic dysregulation, which is often undiagnosed.^41^^,^^42^ Orthostatic hypotension is a common cause of dizziness, unsteadiness, vertigo, and syncope. It is also often associated with postural tachycardia syndrome.^42^^,^^43^ The most common triggers of Orthostatic Dizziness (OD) include routine physical activity, exposure to heat, and postprandial states.^42^

The current diagnostic criteria of OD includes five or more episodes of dizziness, unsteadiness or vertigo triggered by arising (i.e. a change of body posture from lying to sitting/standing or sitting to standing), or present during upright position, which subsides by sitting or lying down), along with documentation of hypotension, tachycardia or syncope on standing or during head-up tilt test.^43^ However, in the suspected cases of “daleyed” variant of postural hypotension, subsequent measurements should be taken.^41^

### Small Vessel Disease (SVD)

SVD appears to be a distinct pathological condition in the elderly, in which even minor BP fluctuations may elicit symptoms.^5^ Although its diagnostic criteria are not well established, SVD is associated with higher levels of cortical microangiopathy rather than classical cardiovascular risk factors.^44^ One hypothesis is that it reflects impaired cerebral autoregulation, particularly in the deep white matter, where cerebral perfusion becomes pressure-dependent and sensitive to mild systemic hypotension, leading to a vague sense of imbalance that does not meet the criteria for other vestibular disorders.^5^

### Blood pressure in sleep conditions

Under normal sleep conditions, blood pressure in healthy individuals follows a circadian pattern, with lower nighttime levels compared to daytime values.^45^ Four circadian BP profiles have been described: (1) Dipping (a 10%–20% nocturnal BP reduction); (2) Nondipping (<10% reduction); (3) Reverse dipping (an increase in nighttime BP); and (4) Extreme dipping (≥20% BP drop).^7^ Nondipping and reverse dipping patterns are associated with increased risk of hypertension-mediated organ damage, cardiovascular events, and all-cause mortality.^46^ In contrast, the clinical and prognostic importance of extreme dipping remains controversial.^7^ Aging and extreme dipping have been reported to be a detrimental combination that leads to cerebrovascular damage and cardiovascular events.^47^ It is plausible that prolonged nocturnal hypoflow may lead to a “low tidal effect,” though supporting evidence remains limited.

### Oxygen delivery

Several pulmonary diseases and severe anemias may also impair oxygen delivery to the VB territory and contribute to the tidal effect^48^, ^49^, ^50^ (Table 1).

## Overlaping situations

However, some diseases can cause VB hypoflow symptoms through siltation and the tidal effect. Furthermore, the occurrence of both phenomena in a patient can be attributed to the coincidental presence of pathological conditions.

### Obstructive sleep apnea (OSA)

OSA is a condition that independently exhibits both mechanisms. Substantial evidence links OSA to a higher incidence and recurrence of stroke.^51^^,^^52^ In OSA, hypoxic episodes trigger inflammation, resulting in a cascade of inflammatory markers, such as IL-1, IL-6, TNF-α, and interferon-γ. These cytokines damage the vascular endothelium and increase platelet aggregation, exacerbating oxidative stress and endothelial injury. This repetitive insult in OSA patients may contribute to stroke. Concurrently, hypoxic events also result in oxygen desaturation, promoting the generation of reactive oxygen species and furthering ischemic injury.^52^

### OSA + Migraine

The link between sleep disorders and migraine is well established. Self-reported poor sleep quality is associated with an increased frequency of migraine attacks,^53^^,^^54^ and preventive migraine treatments may improve sleep quality.^55^

However, the association between migraine and OSA is still controversial. Population-based studies have shown that the prevalence of OSA is similar among individuals with migraine and in the general population without migraine. However, the treatment of OSA with continuous positive airway pressure (CPAP) has been associated with improvements in migraine, including a reduction in the mean frequency of attacks per month (from 5.8 to 0.1 days), mean attack duration, pain intensity, mean number of days with an inability to work, and acute medication intake.^56^

A recent retrospective cohort compared 196,864 adult participants with OSA to a group of 196,864 participants without OSA (1:1 propensity score-matched for age, sex, race, comorbidities, and body mass index categories) in relation to the risk of incident migraine. During the follow-up period, 6.4% and 3.2% of the OSA and non-OSA cohorts developed migraine, respectively. Patients with OSA were found to be 1.85 times more likely to develop migraine (hazard ratio [HR], 1.85; 95% confidence interval [CI], 1.79–1.90).^57^

### Lower blood pressure diseases + VB vessel asymmetries

Another phenomenon explained by this overlapping model is the frequent complaint of spinning vertigo in cardiovascular lower BP diseases, such as OD, heart failure, etc. The spinning sensation is typically generated by a physiological imbalance between the right and left vestibular systems. Hence, Newman-Toker et al. proposed that global reductions in BP lead to local asymmetries in blood flow to the vestibular system because of congenital or acquired left-right asymmetries in vascular caliber, thereby causing vertigo via an ischemic mechanism.^34^^,^^35^

In clinical practice, it has been observed that patients with postural hypotension and VB stenosis are more prone to experiencing episodes of vertigo, darkened vision, and repetitive TIAs.^2^

## Clinical approach

The possibility of VB hypoflow must always be considered in patients who report spontaneous or triggered episodes of dizziness, vertigo, or instability, especially when accompanied by visual darkening, diplopia, syncope, sensory loss, new-onset head or neck pain, trunk ataxia, any observed central nystagmus or central finding in the HINTS+ (Head Impulse, Nystagmus, Test of Skew, and hearing).^1^, ^2^, ^3^

**Fig. 2:**
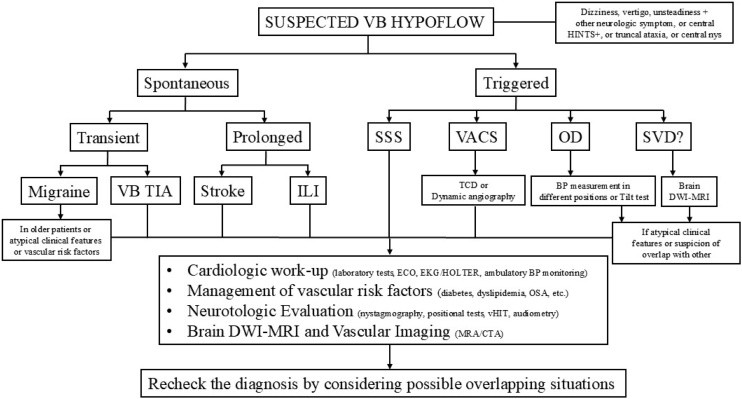
A comprehensive clinical flowchart for diagnosing vertebrobasilar hypoflow. VB, vertebrobasilar; HINTS+, Head Impulse, Nystagmus, Test of Skew, and hearing; nys, nystagmus; SSS, Subclavian Steal Syndrome; VACS, Vertebral Artery Compression Syndrome; OD, Orthostatic Dizziness; SVD, Small Vessel Disease; BP, blood pressure; TIA, transiente ischemic attack; ILI, Isolated Labirinthine Ischemia; TCD, transcranial Doppler ultrasound; DWI-MRI, diffusion-weighted magnetic resonance Imaging; ECO, echocardiography; EKG, electrocardiogram; HOLTER, ambulatory electrocardiographic monitoring; OSA, Obstructive Sleep Apnea syndrome; vHIT, vídeo Head Impulse Test; MRA, magnetic resonance angiography; CTA, computed tomographic angiography.

Several authors have proposed different algorithms and strategies for diagnosing VVD, particularly in emergency departments. A detailed discussion of these is beyond the scope of this review.^58^, ^59^, ^60^, ^61^, ^62^, ^63^, ^64^, ^65^, ^66^, ^67^, ^68^ However, Fig. 2 provides an overview of the clinical reasoning behind diagnosing VVD and the other main causes of vertebrobasilar hypoflow.

The main challenge in cases of transient spontaneous crises is distinguishing vestibular migraine from VB TIA in elderly patients or those with vascular risk factors. The current recommendation is to preemptively perform cardiovascular, neurotologic, and imaging assessments.^60^^,^^61^^,^^64^^,^^68^

The other branch (Fig. 2) includes etiologies that are triggered, such as SSS, VACS, OD, and SVD. Diagnosis of SSS and VACS relies on vascular imaging. Due to the proximity of the involved arteries to the central nervous system and heart, a comprehensive assessment is necessary.^3^^,^^17^, ^18^, ^19^

While there are no established diagnostic criteria for SVD, OD can be proven by BP measurement and the Tilt test.^5^^,^^37^, ^38^, ^39^, ^40^ However, both require the same comprehensive evaluation in cases of atypical presentations and suspected overlapping diseases.

The goal behind the cardiologic, neurotologic, and vascular evaluation is to identify and address avoidable and treatable risk factors, recognize overlapping diseases, and guide medical decision-making.

## Conclusion

When managing a patient presenting with symptoms suggestive of VB hypoflow, clinicians must consider both conditions related to vascular obstruction (the siltation effect) and those originating outside the VB system that nonetheless impair the blood suply (the tidal effect).

Many phenomena still need clarification, such as the extreme dipping, the three-dimensional issues of the VB system architecture, the association of migraine and OSA, and the SVD diagnostic criteria. Undoubtedly, future studies will introduce new concepts and pathophysiological reasoning, refining the current consensus and redefining medical practices in this field.

Therefore, a comprehensive, multidisciplinary clinical evaluation is essential to ensure accurate diagnosis and guide appropriate therapeutic decision-making.

## Funding

This research did not receive any specific grant from funding agencies in the public, commercial, or not-for-profit sectors.

## Data availability statement

The authors declare that all data are available in repository.

## Declaration of competing interest

The authors declare no conflicts of interest.

## References

[1] Grad A., Baloh R.W. (1989). Vertigo of vascular origin: clinical and ENG features in 84 cases. Arch Neurol..

[2] Neto A.C.L., Kim J.S., Bernardo W.M., Bittar R.S.M. (2024). Vertigo and dizziness due to vertebrobasilar TIA: a prospective study. Frontiers Stroke..

[3] Kim J.S., Newman-Toker D.E., Kerber K.A. (2022). Vascular vertigo and dizziness: diagnosticcriteria. J Vestib Res..

[4] Kargiotis O., Siahos S., Safouris A., Feleskouras A., Magoufis G., Tsivgoulis G. (2016). Subclavian steal syndrome with or without arterial stenosis: a review. J Neuroimaging..

[5] Kaski D., Rust H.M., Ibitoye R., Arshad Q., Allum J.H., Bronstein A.M. (2019). Theoretical framework for “unexplained” dizziness in the elderly: the role of small vessel disease. Prog Brain Res..

[6] Neto A.C.L., Bittar R.S.M., Gattas G.S. (2016). Pathophysiology and diagnosis of vertebrobasilar insufficiency: a review of the literature. Int Arch Otorhinolaryngol..

[7] Cuspidi C., Tadic M., Sala C., Gherbesi E., Grassi G., Mancia G. (2019). Extreme dipping: is the cardiovascular risk increased? An unsolved issue. J Hypertens..

[8] Qu M., Liu P., Tao T., Chen Y., Mao L., He X. (2023). Association between vertebrobasilar artery geometry and vertebrobasilar stenosis, recurrence, and death in posterior circulation stroke and transient ischemic attack. J Stroke Cerebrovasc Dis..

[9] Katsanos A.H., Giannopoulos S. (2017). Increased risk for posterior circulation ischaemia in patients with vertebral artery hypoplasia: a systematic review and meta-analysis. Eur Stroke J..

[10] Wake-Buck A.K., Gatenby J.C., Gore J.C. (2012). Hemodynamic characteristics of the vertebrobasilar system analyzed using MRI-based models. PLoS One..

[11] Kobayashi N., Karino T. (2010). Flow patterns and velocity distributions in the human vertebrobasilar arterial system. J Neurosurg..

[12] Krijger J.K.B., Berend H., Hendrik W.H. (1989). Mathematical models of the flow in the basilar artery. J Biomech..

[13] Rutledge K., McDaniel M., Teng S., Hall H., Ramroop T., Sprout E., et al. From: https://education.nationalgeographic.org/resource/tidal-bore/ in Jun, 15th 2025.

[14] Kleindorfer D.O., Towfighi A., Chaturvedi S. (2021). 2021 guideline for the preventionof stroke in patients with stroke and transient ischemic attack: aguideline from the American Heart Association/American StrokeAssociation. Stroke..

[15] Liqun Z., Park K.H., Kim H.J., Lee S.U., Choi J.Y., Kim J.S. (2018). Acute unilateral audiovestibulopathy due to embolic labyrinthine infarction. Front Neurol..

[16] Strickland B.A., Pham M.H., Bakhsheshian J., Russin J.J., Mack W.J., Acosta F.L. (2017). Bow Hunter’s syndrome: surgical management (video) and review of the literature. World Neurosurg..

[17] Alcocer F., David M., Goodman R., Jain S.K.A., David S. (2013). A forgotten vascular disease with important clinical implications. Subclavian steal syndrome. Am J Case Rep..

[18] Osiro S., Zurada A., Gielecki J. (2012). A review of subclavian steal syndrome with clinical correlation. Med Sci Monit..

[19] Chen S.P., Hu Y.P., Fan L.H., Zhu X.L. (2013). Bidirectional flow in the vertebral artery is not always indicative of the subclavian steal phenomenon. J Ultrasound Med..

[20] Choi K.D., Jo J.W., Park K.P. (2007). Diffusion-weighted imaging of intramural hematoma in vertebral artery dissection. J Neurol Sci..

[21] Kim B.M., Kim S.H., Kim D.I. (2011). Outcomes and prognostic factors of intracranial unruptured vertebrobasilar artery dissection. Neurology..

[22] Huh G., Bae Y.J., Woo H.J., Park J.H., Koo J.W., Song J.J. (2020). Vestibulocochlear symptoms caused by vertebrobasilar dolichoectasia. Clin Exp Otorhinolaryngol..

[23] Yuan Y.J., Xu K., Luo Q., Yu J.L. (2014). Research progress on vertebrobasilar dolichoectasia. Int J Med Sci..

[24] Ubogu E.E., Zaidat O.O. (2004). Vertebrobasilar dolichoectasia diagnosed by magnetic resonance angiography and risk of stroke and death: a cohort study. J Neurol Neurosurg Psychiatry..

[25] Smoker W.R., Price M.J., Keyes W.D., Corbett J.J., Gentry L.R. (1986). High-resolution computed tomography of the basilar artery: 1. Normal size and position. Am J Neuroradiol.

[26] Rodríguez C.B.A., Ugalde D.M.M., García-Tecpa R.A., Rodríguez C.B.A. (2022). Case Report: mixed-cause vertigo and sudden sensorineural hearing loss as presentations of vertebrobasilar dolichoectasia. Cureus.

[27] Prasad S.N., Singh V., Selvamurugan V., Phadke R.V. (2021). Vertebrobasilar dolichoectasia with typical radiological features. BMJ Case Rep..

[28] Frosolini A., Fantin F., Caragli V. (2023). Vertebrobasilar and basilar dolichoectasia causing audio-vestibular manifestations: a case series with a brief literature review. Diagnostics..

[29] Leppert M.H., Poisson S.N., Scarbro S. (2024). Association of traditional and nontraditional risk factors in the development of strokes among young adults by sex and age group: a retrospective case-control study. Circ Cardiovasc Qual Outcomes..

[30] Zhang Y., Parikh A., Qian S. (2017). Migraine and stroke. Stroke Vasc Neurol..

[31] Borończyk M., Zduńska A., Węgrzynek-Gallina J., Grodzka O., Lasek-Bal A., Domitrz I. (2025). Migraine and stroke: correlation, coexistence, dependence-a modern perspective. J Headache Pain..

[32] Calic Z., Cappelen‐Smith C., Zagami A.S. (2015). Reversible cerebral vasoconstriction syndrome. Int Med J..

[33] Chen S.P., Wang S.J. (2022). Pathophysiology of reversible cerebral vasoconstriction syndrome. J Biomed Sci..

[34] Newman-Toker D.E., Dy F.J. (2008). How often is dizziness from primary cardiovascular disease true vertigo? A systematic review. J Gen Intern Med..

[35] Newman-Toker D.E., Camargo C.A. (2006). ‘Cardiogenic vertigo’—true vertigo as the presenting manifestation of primary cardiac disease. Nat Clin Pract Neurol..

[36] Linzer M., Pritchett E.L.C., Pontinen M., McCarthy E., Divine G.W. (1990). Incremental diagnostic yield of loop electrocardiographic recorders in unexplained syncope. Am J Cardiol..

[37] Sivakumaran S., Krahn A.D., Klein G.J. (2003). A prospective randomized comparison of loop recorders versus Holter monitors in patients with syncope or presyncope. Am J Med..

[38] Bollmann A., Husser D., Stridh M. (2007). Atrial fibrillatory rate and risk of left atrial thrombus in atrial fibrillation. Europace..

[39] Sharma S., Hashmi M.F., Bhattacharya P.T. (2023).

[40] Sari N., Jaehde U., Wermund A.M. (2025). Identification of potentially causative drugs associated with hypotension: a scoping review. Archiv der Pharmazie..

[41] Ricci F., De Caterina R., Fedorowski A. (2015). Orthostatic hypotension: epidemiology, prognosis, and treatment. J Am Coll Cardiol..

[42] Low P.A., Opfer-Gehrking T.L., McPhee B.R. (1995). Prospective evaluation of clinical characteristics of orthostatic hypotension. In Mayo Clinic Proceedings.

[43] Kim H.A., Bisdorff A., Bronstein A.M. (2019). Hemodynamic orthostatic dizziness/vertigo: diagnostic criteria: consensus document of the committee for the classification of vestibular disorders of the Bárány Society. J Vestib Res..

[44] Ahmad H., Cerchiai N., Mancuso M., Casani A.P., Bronstein A.M. (2015). Are White matter abnormalities associated with “unexplained dizziness”?. J Neurol Sci..

[45] Mancia G., Verdecchia P. (2015). Clinical value of ambulatory blood pressure, evidence and limits. Circ Res.

[46] Ohkubo T., Hozawa A., Yamaguchi J. (2002). Prognostic significance of nocturnal decline in blood pressure n subjects with and without high 24-h blood pressure: the Ohasama study. J Hypertens..

[47] Kario K., Pickering T.G., Matsuo T., Hoshide S., Schwartz J.E., Shimada K. (2001). Stroke prognosis and abnormal nocturnal blood pressure falls in older hypertensives. Hypertension..

[48] Ludwig H., Strasser K. (2001).

[49] Hughes J.D., Rubin L.J. (1986). Primary pulmonary hypertension: an analysis of 28 cases and a review of the literature. Medicine.

[50] Li J., Liao Y., Li J., Liu F. (2021). Lung cancer with dizziness as the initial symptom: a case report and literature review. Chin General Pract..

[51] Javaheri S., Peker Y., Yaggi H.K., Bassetti C.L. (2022). Obstructive sleep apnea and stroke: the mechanisms, the randomized trials, and the road ahead. Sleep Med Rev..

[52] Jehan S., Farag M., Zizi F. (2018). Obstructive sleep apnea and stroke. Sleep Med Dis Int J.

[53] Song T.J., Yun C.H., Cho S.J., Kim W.J., Yang K.I., Chu M.K. (2018). Short sleep duration and poor sleep quality among migraineurs: a population-based study. Cephalalgia..

[54] Beh S.C. (2019). Vestibular migraine: how to sort it out and what to do about it. J Neuroophthalmol..

[55] Aydinlar E.I., Dikmen P.Y., Kosak S., Kocaman A.S. (2017). OnabotulinumtoxinA effectiveness on chronic migraine, negative emotional states and sleep quality: a single-center prospective cohort study. J Headache Pain..

[56] Tiseo C., Vacca A., Felbush A., European Headache Federation School of Advanced Studies (EHF-SAS) (2020). Migraine and sleep disorders: a systematic review. J Headache Pain.

[57] Chen T.Y.T., Hsieh T.Y.J., Wang Y.H., Chang R., Hung Y.M., Wei J.C.C. (2025). Association between obstructive sleep apnea and migraine: a United States population‐based cohort study. Headache..

[58] Kattah J.C., Talkad A.V., Wang D.Z., Hsieh Y.H., Newman-Toker D.E. (2009). HINTS to diagnose stroke in the acute vestibular syndrome: three-step bedside oculomotor examination more sensitive than early MRI diffusion-weighted imaging. Stroke..

[59] Vanni S., Pecci R., Edlow J.A. (2017). Differential diagnosis of vertigo in the emergency department: a prospective validation study of the STANDING algorithm. Front Neurol..

[60] Edlow J.A., Carpenter C., Akhter M. (2023). Guidelines for reasonable and appropriate care in the emergency department 3 (GRACE‐3): acute dizziness and vertigo in the emergency department. Acad Emerg Med..

[61] Filippopulos F.M., Strobl R., Belanovic B. (2022). Validation of a comprehensive diagnostic algorithm for patients with acute vertigo and dizziness. Eur J Neurol..

[62] Ahmadi S.A., Vivar G., Navab N. (2020). Modern machine-learning can support diagnostic differentiation of central and peripheral acute vestibular disorders. J Neurol.

[63] Kuroda R., Nakada T., Ojima T. (2017). The TriAGe+ score for vertigo or dizziness: a diagnostic model for stroke in the emergency department. J Stroke Cerebrovasc Dis..

[64] Newman-Toker D.E., Edlow J.A. (2015). TiTrATE: a novel, evidence-based approach to diagnosing acute dizziness and vertigo. Neurol Clin..

[65] Koohi N., Male A.J., Kaski D. (2023). Acute positional vertigo in the emergency department—peripheral vs. central positional nystagmus. Front Neurol..

[66] Sadok N., Luijten G., Bahnsen F.H. (2025). Performing the HINTS-exam using a mixed-reality head-mounted display in patients with acute vestibular syndrome: a feasibility study. Front Neurol..

[67] Cortese E., Rochelle P.L., Patel F., Koohi N., Kaski D. (2025). Integrated diagnostic algorithm for acute vertigo combining TiTrATE, STANDING, and HINTS: a validation study in the emergency department. Sci Rep..

[68] Edlow J.A., Bellolio F. (2024). Recognizing posterior circulation transient ischemic attacks presenting as episodic isolated dizziness. Ann Emerg Med..

